# Effects of cycling workstation to get tertiary employee moving on their overall health: study protocol for a REMOVE trial

**DOI:** 10.1186/s13063-021-05317-2

**Published:** 2021-05-22

**Authors:** Terry Guirado, Lore Metz, Bruno Pereira, Audrey Bergouignan, David Thivel, Martine Duclos

**Affiliations:** 1 EA 3533, Laboratory of the Metabolic Adaptations to Exercise under Physiological and Pathological Conditions (AME2P), UE3533, Clermont Auvergne University, F-63171 63170 Aubiere CEDEX, 80026 Clermont-Ferrand, BP France; 2 Auvergne Research Center for Human Nutrition (CRNH), 63000 Clermont-Ferrand, France; 3 Department of Sport Medicine and Functional Explorations, Clermont-Ferrand University Hospital, G. Montpied Hospital, Clermont-Ferrand, France; 4 INRA, UMR 1019, Clermont-Ferrand, France; 5 Clermont-Ferrand University Hospital, Biostatistics Unit (DRCI), Clermont-Ferrand, France; 6 Université de Strasbourg, CNRS, IPHC UMR 7178, F-67000 Strasbourg, France; 7 Division of Endocrinology, Metabolism and Diabetes, Anschutz Health & Wellness Center, University of Colorado, Anschutz Medical Campus, Aurora, CO USA

**Keywords:** Sedentary behaviours, Physical activity, Cycling workstation, Randomized controlled trial, Workplace, Tertiary societies, Prevention

## Abstract

**Background:**

Sedentary behaviour (SB) and low levels of physical activity (PA) are predictors of morbidity and mortality. Tertiary employees spend a considerable amount of their daily time seated and new efficient strategies to both reduce sedentary time and increase physical activity are needed. In that context, the REMOVE study aims at evaluating the health effects of a 24-week cycling desk intervention among office workers.

**Methods:**

A prospective, open-label, multicentre, two-arm parallel, randomized controlled trial (RCT) will be conducted in office-sitting desk workers. Office workers (*N* = 80) who have 0.8 full time equivalent hours (FTE) and 75% of this time in a sitting position will be recruited from tertiary worksites in Clermont-Ferrand, France. Subjects will be randomly assigned to one of the two following interventions: (i) PPM6: performance of two 30 min of cycling desk (using portable pedal exercise machine—PPM) per working day for 6 months or (ii) CTL_PPM3: 3 months with no intervention (control) followed by 3 months during which workers will be asked to complete two 30 min of PPM per working day. At baseline (T0), at 3 months (T1) and at 6 months (T2) after the start of the interventions, primary outcomes; 7-day PA and SB (3D-accelerometers), secondary outcomes; body composition (bioelectrical impedance), physical fitness (aerobic fitness, upper and lower limb strength), metabolic outcomes (fasting blood samples), self-perceived stress, anxiety, quality of life at work and job strain (questionnaires), tertiary outcomes; resting metabolic rate and cycling energy expenditure (indirect calorimetry) and eating behaviours (questionnaires) will be measured. An ergonomic approach based on observations and individual interviews will be used to identify parameters that could determine adherence.

**Discussion:**

The REMOVE study will be the first RCT to assess the effects of cycling workstations on objectively measured PA and SB during working and non-working hours and on key physiological and psychological health outcomes. This study will provide important information regarding the implementation of such cycling workstations in office workers and on the associated potential health benefits.

**Trial registration:**

ClinicalTrials.govNCT04153214. Registered on November 2019, version 1

## Background

Sedentary behaviour (SB) is defined as any waking behaviour characterized by an energy expenditure ≤ 1.5 metabolic equivalents (METs), while in a sitting, reclining or lying posture [1, 2]. Time spent sedentary and low levels of physical activity (PA) are known to be major predictors of cardiometabolic risks [3, 4]. Epidemiological studies further suggest that people engaged in prolonged (uninterrupted) have higher cardio-metabolic risks than people who regularly break up their sitting time, independent of the total time spent sitting and physically active [5, 6].

SB has continuously increased over the last century due to environmental changes, including technology, structure of industries, automation and computerisation [7–9]. With the emergence and expansion of the tertiary activities, the occupation domain has largely contributed to the general adoption of SB, especially with the generalization of the desk-bound activities [10]. For instance, during an 8-h working day, office workers spend on average about 77% of the time sitting, 18% standing and only 5% in other activities [11]. Office workers are therefore particularly vulnerable to the adverse health effects of SB.

Thus, the workplace represents an ideal setting to implement strategies to promote PA and reduce SB, especially prolonged SB, and thus improve employee’s health [12, 13]. Recently, various strategies to reduce sedentary time in the workplace have been developed [14]. The use of active workstations such as desks coupled with a treadmill, a cycling station or step have been reported to reduce sedentary time at work and increase PA [15] along with positive health effects [16]. Most of these studies were conducted in individuals suffering from overweight or obesity and tested whether such workstations were efficient to increase daily energy expenditure. However, data in normal weight people to support the preventative effect of such strategies remain scarce [17]. In addition, the long-term effectiveness of programs using active workstations to increase PA and reduce SB on time spent sedentary (total duration, average duration of each bout, number of breaks, etc.) and physically active but also on key biological parameters have not been determined yet [18, 19]. Assessing the effect of such a strategy on physical fitness is particularly relevant given that both endurance capacity [20–22] and muscle strength [23] are powerful predictors of mortality and morbidity independent of the health status of the individuals.

Interventions should not only focus on the operational feasibility but also consider the potential side-effects on the employees’ work task quality and productivity. Interventions aiming at increasing PA and reducing SB in office workers should assess the coherence and compatibility with the working environment, structuration, culture and the way they live and experience their work [24].

To date, results on the effects of interventions in the work environment to reduce SB are not clear-cut, potentially because of the limited number of studies, the relatively low samples sizes, the heterogeneity in the study designs and the lack of highly rigorous studies. Based on this observation, Shrestha et al. concluded on the need for long-term, multi-place, randomized controlled studies with objective measurement of PA and SB not only during the work period but also outside of work [19]. On top of these methodological issues is the use of treadmills or cycling desks in most of the available studies; the implementation of these devices is however limited because of their high cost, large size (that may not fit in small offices) and lack of portability [25]. Portable pedal machine (PPM) have been recently designed and proposed for such interventions. They can be settled under a desk, sparing a lot of space, and are easy to use. They open new possibilities for the development of strategies aiming at fighting against SB in tertiary worksite [16, 25].

## Objectives

The primary objective of this study is to evaluate the effects of a cycling workstation program (30 min twice a day) for 3 months among tertiary employees on PA and SB during working and non-working days. Our secondary objective is to assess the effect of this cycling workstation program on body composition, physical fitness and cardiometabolic risk factors (composite measure of fasting glucose, insulin, triglycerides, HDL-cholesterol, LDL-cholesterol, C-reactive protein or CRP, blood pressure and heart rate variability) and psychological parameters. Our third objective is to do an ergonomic assessment and determine the effects of this cycling workstation program on resting metabolic rate, cycling energy expenditure, eating behaviour and the implementation of the device in the worksite. Finally, we will extend the initial 3-month intervention with 3 additional months and thus test the 6-month effects of the intervention on the above-mentioned outcomes.

## Hypothesis

We hypothesize that the daily use of a cycling workstation for 3 months will increase PA and reduce SB on both working and non-working days and will improve physical and psychological health outcomes compared to a control condition with no intervention. The addition of three extra months of intervention (i.e. 6 months total) will further improve the study parameters compared to the effects that will be observed after 3 months of intervention.

## Methods and analysis

### Study design

**Fig. 1:**
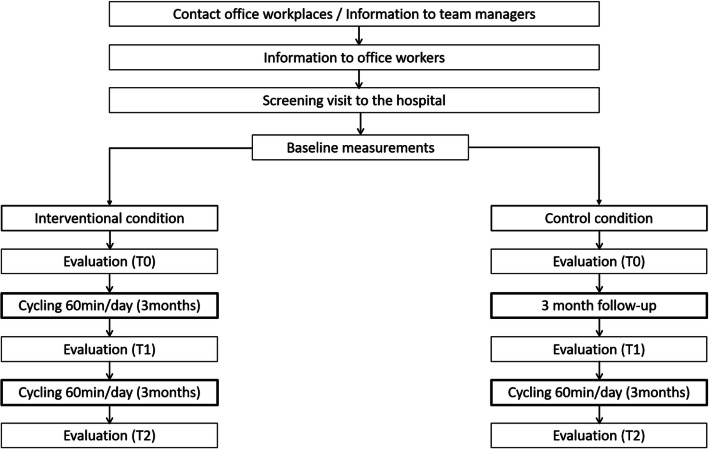
Study overview

The REMOVE study is a prospective, exploratory, randomized, controlled, open-label, multicentre, two-arm parallel trial in office-sitting desk workers (Fig. 1).

Eighty tertiary employees from Clermont-Ferrand (France) will be recruited to participate in this protocol. Three worksites of moderate size (*n* = 80 employees across the 3 sites) will be randomly assigned to one of the two interventions: (1) CTL_PPM3, an intervention with two consecutive periods: 3 months with no change of working conditions (CTL: control phase) and 3 months of PPM use (PPM3), and (2) PPM6, an intervention with 6 months of PPM use. Tests will be performed on both groups at baseline (T0), after 3 (T1) and 6 months (T2) of the intervention. Information sheets will be given to all the participants and written informed consent will be obtained for each participant. This study has been approved by the ethical authorities (CPP IDF VIII 19 09 66) and registered as a clinical trial under the reference number NCT04153214 (clinicaltrials.gov).

### Study setting and eligibility

Worksites will be recruited in Clermont-Ferrand, Region Rhones-Alpes-Auvergne, France. Selected worksites will be drawn from distinct work sectors: administration of the Universities, healthcare administration and school board.

### Study eligibility

#### Worksite eligibility criteria

Worksites will be selected if they respond to the following criteria: desk-bound job employees, no use of sit-stand desk or other active working station, every CEO agrees that participants will do the three test sessions (2 h each) during working hours. The worksites will not interact between each other.

#### Participant eligibility criteria

Participation is voluntary. Participants will be recruited if they fulfil the following criteria: aged 18–61 years, BMI ≤ 29.9 kg/m^2^, are employed more than 80% full time with 75 % of this time in a sitting position (seated office work such as computer or telephone-based work), do not have a contact job where the PPM can be badly experienced by the visitor, do not practice PA more than 2.5 h per week, are able to cycle for 30-min per session and 60-min per day and agree to be randomized.

#### Participant exclusion criteria

Employees will not be included if they have acute or chronic infections, oncological diseases, joint replacements or any surgery within the previous 6 weeks, psychiatric disorders, are pregnant or breastfeeding women, have diabetes treated with insulin and any condition that might affect the use of the PPM.

### Protocol status

#### Recruitment of participants

At the selected sites, an informal session will be conducted among employees, detailing the objectives of the study and the protocol. Following this first meeting, volunteers will inform their managers and the investigators of their willingness to participate. Then, they will have a screening visit with a member of the study team to verify inclusion and exclusion criteria and sign a written informed consent. Prior to any inclusion, study requirements will be repeated and participants will be able to withdraw from the study at any time without giving any explanation.

#### Randomization

A permuted-block randomization (i.e. random block sizes) will be conducted by the study biostatistician using a computer-generated random allocation (Stata software, version 15, StataCorp, College Station, US), with a 1:1 ratio allocation, ensuring complete randomness of the assignment of a patient to each randomized group. The randomization will be stratified by centre. The randomization and allocation process will be performed by the statistician involved in this study. Physicians will be involved for the enrolment participants.

### Intervention

The objective of the study is to replace 30 min of sedentary time by 30 min of PA of light intensity (LPA), twice a day, during the five working days (i.e. 60 min/day). Therefore, office workers should accumulate 5 h of LPA per week. This study design choice was motivated by recent observations from epidemiological studies. The replacement of 60 min per day of sedentary time by LPA has been associated with a decrease in the risk of all-cause mortality by 40% in a study [26] and 18 % in another one [27].

Volunteers will be provided PPM (DeskCycle, 3D Innovations LLC., Greeley, CO, USA). The device requires only little space and can be slipped under the desk while using it or not.

Subjects enrolled in PPM6 group will be equipped with a PPM under their desk and will use it 60 min per working day (30 min continuously in the morning and 30 min in the afternoon) during 6 months.

Subjects in the CTL_PPM3 group will maintain their habitual daily activities during the first 3 months. Then, they will also have a PPM under their desk and will use it 60 min per working day (30 min in the morning and 30 min in the afternoon) during the next 3 months. The time of use in the morning or afternoon is left to the choice of the subjects and may change from one day to another.

Before using the PPM, every participant will have a familiarization visit for the use of the device. Thus, if needed, their workstation will be adapted to adjust their seat with the Deskcycle. While the PPM proposes 8 levels of magnetic resistance, all the participants will perform their cycling exercises at the same intensity (resistance level 2) that allows to pedal with a minimum of resistance. The speed will be chosen by participants to not disturb them during their work. PPM has a monitor to inform users about the completed total distance, duration of exercise, speed of cycling and revolutions per minute (RPM). Subjects will fill up a notebook on a Microsoft Excel files to inform investigators of what he/she did at each session of cycling (performed : yes or no, completed distance, time and duration of cycling) that will be gathered every week by the study team. This information will be used to estimate participants’ adherence to the intervention.

Notebooks will inform us if participants properly completed the intervention. Pop-up reminders will be sent via emails to the participants twice a day (1 h before lunch break and 1 h before the end of daywork) and will contain information regarding the overall effects of sedentary time on health and on the benefits of PA. Pop-up notifications will be used if participants will not reach three times in a working week the 30 min per half-day. Such motivational approach has been shown to be efficient and will be used here to motivate participants to maintain the requested 60 min of cycling per day [25]. Every week, a member of the research team will meet the participants at their workplace to have direct feedback on their use of the PPM and to keep their level of engagement as high as possible. Each participant should keep their daily activities and care without any modification except those asked in the protocol.

### Data collection

All measurements will be performed on three different occasions: baseline (T0), 3 months (T1) and 6 months (T2). Figure 1 and Table 1 present the overall study. All the clinical measurements will be done at the Laboratory of the Metabolic Adaptations to Exercise under Physiological and Pathological Conditions, in Clermont-Ferrand (France).

**Table 1:** Measurements timeline for both groups

Measurements timeline for both groups			
Measurement	Baseline	3 months	6 months
Socio-demographic data	X		
Objectively measured of PA and SED	X	X	X
Self-reported lifestyle behaviours	X	X	X
Health-related outcomes	X	X	X
Body composition	X	X	X
Physical fitness	X	X	X
Metabolic outcomes	X	X	X
Stress and anxiety	X	X	X
Life span and quality	X	X	X
Resting metabolic rate	X	X	X
Satisfaction and adherence			X

### Socio-demographic data

At baseline, socio-demographic data including age, gender, education level, marital status, household composition, number of children living in the household and employment status (i.e. length of tenure, working hours, overtime hours and job classification) will be collected. Also, medications and/or altered doses of an existing treatment over the last 3 months will be recorded. At T1 and T2, participants will be asked to report any life events that may have occurred over the past 3 months that may have affected their health or wellbeing.

### Primary outcomes

#### Physical activity level and sedentary time

Two devices will be used, to ensure a more precise evaluation of PA [28]. Daily light, moderate, and vigorous PA will be measured objectively with the tri-axial Actigraph GT3x-BT monitor (AG) (Actigraph LLC, Pensacola, Fl, USA). This monitor will be worn at the waist above the right hip with an elastic belt during 5 days [29]. Participants will be asked to wear AG during all their waking hours, except during water-based activities. This device is accurate to classify PA in daily life environments [30] and have been validated in adult population [31].

The second device used is an activPAL3 micro (AP) (PAL Technologies Limited, Glasgow, UK; default settings) worn on the mid-line of the right thigh. This inclinometer monitor will be worn continuously during the same 5 days than the AG except during water-based activities. ActivPAL3 micro has been validated to evaluate time spent sitting/lying, standing, walking, number of sedentary bouts and number of breaks of those sedentary bouts [32, 33]. Participants will be asked to precisely indicate when they will use the PPM since AP might not be sensitive enough to detect this behaviour [34].

During each measurement period, subjects will report their exact bed time, wake up time, time they start wearing the activity monitors and time when they took them of. Participants will also be asked to report the time they arrive at work and leave from work as well as any period of not wearing the monitors. This information will be used to calculate the variables from the activity devices during the entire day on both working and non-working days. Time spent active and sedentary will also be assessed during the working hours and non-working hours of the working days.

### Secondary outcomes

#### Health-related outcomes

Blood pressure will be measured three times at each visit on the right arm of the participant who will be in a seated position. Only the last value will be reported.

For anthropometric measurements, participants will wear light indoor clothing without shoes. Body height will be assessed with a stadiometer. Value will be taken to the closest 0.1 cm. Body weight will be assessed to the closest 0.1 kg with a scale (Tanita MC-780, Tanita, USA, Arlington Heights). Thereby, body mass index will be calculated using following formula BMI = weight(kg)/ height(m)^2^.

Waist circumference (WC) will be measured with a tape measure to the nearest 0.5 cm, and WC to height ratio (WHtR) will be calculated. This ratio is a better predictor of intra-abdominal fat mass than WC alone [35] in both men and women [36].

#### Body composition

FM distribution, assessed by WHtR, and total FM are good predictors of cardiovascular disease [37], cardiometabolic disease [38] and some cancers including colorectal [39, 40], prostate [41], liver [42] and breast [43] cancers.

FFM has a high metabolic activity, plays a major role in metabolism (whole-body protein metabolism [44], post-prandial glucose uptake) and is the main determinant of resting energy expenditure [45].

Body composition will be evaluated in fasting conditions with a bioelectrical impedance (Tanita MC-780, USA, Arlington Heights) to determine absolute values of fat mass (FM) and fat-free mass (FFM). This device has shown a good reliability to obtain FM and FFM for adults [46] with different levels of PA [47] compared to measurements with dual-energy X-ray absorptiometry (DXA), considered to be the gold-standard method.

#### Physical fitness

Systematic review and meta-analysis reported that a low cardiorespiratory fitness is associated with higher risks of morbidity and mortality for people in good health [48, 49] or with chronic disease [50, 51]. Reduced muscle strength, as measured by grip strength, has also been associated with an increased risk of all-cause and cardiovascular mortality in socio-culturally and economically diverse countries [52].

Aerobic fitness will be evaluated with the Step Test. The step used for this test will be at a height of 16.25 inches (41.3 cm) for men and 11.8 inches (30 cm) for women. Stepping up and down will last for 6 min at the rate of 24 cycles per minute, set by a metronome. Subjects will wear a heart rate monitor Zephyr™ BioHarness™ (Zephyr Technology, Annapolis, USA) during the test. Heart rate will be recorded at the end of the test, after 30 s and 60 s of recovery. This test has been found reliable for healthy adults and is highly reproducible [53].

Low muscle mass and strength is associated with higher risk of mortality [54]. Muscle strength will be evaluated through upper and lower limb strength. A handgrip dynamometer will be used with dominant hand to assess upper limb strength. Three measurements will be made and only the best one will be registered. This method has already proved its efficiency and reliability with healthy adults [23, 55, 56] and in clinical use [57].

Lower limb strength will be assessed through a knee-extension power test on an isokinetic dynamometer (Biodex System 2, Biodex, Shirley, USA). Torque and power data will be corrected for gravity and will be digitized and exported at a rate of 2 kHz to an external data acquisition system (PowerLab 8/35; ADInstrument, New South Wales, Australia) driven by the LabChart 7.3 Pro software (ADInstrument, New South Wales, Australia). This isokinetic dynamometer has been validated and found reliable for isometric and concentric assessment [58, 59]. Subjects will be seated comfortably with a hip joint at 105° of flexion and will be attached on the trunk, the hip and the left leg to the dynamometer chair with Velcro straps to provide a complete stability during every maximal contraction. The dynamometer lever arm will be attached to the right leg by a strap positioned 1–2 cm above the lateral malleolus. Torque will be measured at a knee joint angle of 90°. Participants will perform three isometric 3 s-Maximum Voluntary Contraction (MVC) with 2 min of rest between each try. Peak torque values will be checked during data collection and an additional test will be done when there is a torque variation higher than 10% between the three tests. Then, only the best one will be reported.

Concentric torque will also be measured during three MVCs performed at a velocity of 60°/s and 120°/s. The extension will be at their maximal abilities when the flexion will be done at their own comfort speed. Between the two different velocities, subject will have 2 min of rest. Only the best peak power will be reported.

#### Metabolic outcomes

Large volumes of SB sedentary time increase cardiometabolic risks and inflammatory biomarkers [60]. Reducing SB with an increasing of PA are more favourable to preserve metabolic health [61, 62].

Basal blood samples will be drawn after a 12-h overnight fast by a nurse. It will be asked to every subject to keep their usual daily activities. However, they will be instructed to avoid high intensity exercise and stressful situations on the day before the test and to avoid caffeine consumption and nicotine in the morning before the test.

Blood will be centrifuged and plasma will be kept frozen in aliquots at − 80 °C until analysis in batch. All samples will be shipped to laboratories on dry ice at each end of evaluation period (T0, T1, T2).

Lipids profile and inflammatory markers will be measured using a Dimension Vista® system (Siemens Healthcare GmbH, Erlangen, Germany). Plasma triglycerides will be measured with Dimenson Flex™ reagent cartridges (inter-assay CV = 3% at 68 mg/dL, 2% at 384 mg/dL) (intra-assay CV = 4% at 68 mg/dL, 2% at 384 mg/dL). Plasma LDL-cholesterol will be assessed with Dimenson Flex™ reagent cartridges (inter-assay CV = 2% at 50 mg/dL, 2% at 145 mg/dL) (intra-assay CV = 4% at 50 mg/dL, 4% at 145 mg/dL). Plasma HDL-cholesterol will be measured with Dimenson Flex™ reagent cartridges (inter-assay CV = 1.6% at 47 mg/dL, 1.9% at 69 mg/dL) (intra-assay CV = 2.3% at 47 mg/dL, 2.1% at 67 mg/dL). Plasma glucose will be measured with Dimenson Flex™ reagent cartridges (inter-assay CV = 2% at 75 mg/dL, 1% at 379 mg/dL) (intra-assay CV = 3% at 75 mg/dL, 2% at 379 mg/dL). Plasma insulin will be assessed with IMMULITE® 2000 System (inter-assay CV = 7.3% at 7.67 μIU/mL, 5% at 26.4 μIU/mL) (intra-assay CV = 5.5% at 7.67 μIU/mL, 3.9% at 26.4 μIU/mL). Insulin resistance will be determined with the use of the homeostasis model assessment of insulin resistance (HOMA-IR) [63]. Plasma high sensitivity C-reactive protein will be measured with Dimenson Flex™ reagent cartridges (inter-assay CV = 4.8% at 0.115 mg/dL, 5.4% at 0.346 mg/dL) (intra-assay CV = 4% at 0.115 mg/dL, 4.4% at 0.346 mg/dL).

#### Stress and anxiety

High levels of SB have been associated with increased risks for mental disturbances [64] such as stress [65], depression [66, 67] or anxiety [68]. On the other hand, meta-analysis demonstrated that regular PA decreases anxiety [69] and depression [70]. Stress and anxiety will be measured by questionnaires and objective methods (blood anandamide levels and heart rate variability). Plasma N-arachidonylethanolamine, also known as anandamide (AEA), is a lipid which is involved in pain perception, anxiety and depression and in stress response [71]. It has been shown that anxiety inversely correlates with blood AEA levels in humans [72, 73]. Blood AEA will be measured by enzyme-linked immunosorbent assay (ELISA) (Clound-Clone Corporation, Houston, TX, USA) (inter-assay CV < 12%, intra-assay: CV < 10%; detection range 2.47–200 ng/mL).

Heart rate variability is a cardiovascular risk marker [74] associated with burnout [75] and chronic stress [76]. It is also an indirect measure of autonomic nervous system [77, 78]. It will be evaluated during 24 h with heart rate monitor Zephyr™ BioHarness™ (Zephyr Technology, Annapolis, USA), and analysed according to the “Task Force recommendations” [79].

Stress level at work [80] and at home will be assessed by a visual analogic scale (VAS) on a horizontal line of 100 mm, ranging from very low (1—no stress) to very high (100—maximal stress).

Equanimity is the ability to have an attentional, emotional and cognitive balance of the mind [81]. This parameter will be evaluated with the Two Factor Equanimity Scale (EQUA-S), which has been validated [82].

#### Quality of life at work and job strain

A socio-ecological approach suggests to evaluate individual behaviour with a multiple level factors focused on the social and physical environment [83]. Reducing SB with an increasing of moderate-to-vigorous PA can be a strategy to improve the quality of life (QOL) [84]. To our knowledge, no study has assessed the effect of increasing light PA and reducing SB on the worksite on QOL.

The Job Content Questionnaire is one of the most widely used questionnaires; it relates the characteristics of a workplace to health and well-being [85] and relies on the demand-control model [86]. This test has shown a good reliability with middle-aged adults [87]. The imbalance between high efforts spent at work and low rewards received will be assessed by the effort-reward imbalance (ERI) questionnaire designed by Siegrist [88]. This questionnaire has also been validated in this population [89].

Pain will be evaluated by the Nordic Musculoskeletal Questionnaire (NMQ) [90, 91]. This questionnaire has been validated in working population [92] including office workers [93].

To evaluate the QOL at work, five different VAS for mental and physical fatigue, burnout, life and sleep quality will be used. These VAS will be on a horizontal line of 100 mm, ranging from very low (1) to very high (100) [77].

### Exploratory outcomes

#### Resting metabolic rate and cycling energy expenditure

Resting metabolic rate (RMR) will be measured by indirect calorimetry (FitMate™ -COSMED, Rome, Italy) [94]. This method with FitMate™ device has been validated in healthy adults [95]. Before the test, gas analysis will be calibrated following the instructions of the manufacturer’s recommendations. Subjects will stay 20 min in a chair and remain calm in a quiet place. O2 consumption will be measured for 15 min minimum and the last 5 min will be reported [96]. RMR will be calculated using the Weir’s equation [97].

Participants will undergo a 30-min exercise testing using the DeskCycle device. The objective will be to demonstrate that this exercise is of light-intensity. It will also allow to observe whether there is a metabolic adaptation after 3 and 6 months of using the PPM. During the exercise testing, energy expenditure and substrate utilization will be calculated by measuring gas exchange by indirect calorimetry with a portable device (MetaMax 3b; Cortex Biophysik, Leipzig, Germany). This device has been validated for gas exchange measurements during exercise [98]. Before each test, gas analysis will be calibrated according to the manufacturer’s recommendations. Subject will be seated in a comfortable position to pedal and in a thermoneutral environment. The resistance will be set at 2 and RPM at 50 during the whole test. Gas exchange will be measured for 30 min. Energy expenditure will be derived from calorimetry-measured VO2 and VCO2 using Weir’s equation [97]. Substrate utilization will be derived using Peronnet and Massicotte equation [99].

#### Eating behaviours

Modern sedentary behaviours are known to promote food overconsumption [100, 101], which coupled with low levels of energy expenditure can favour chronic positive energy balance and long-term weight gain. The effect of the intervention on eating behaviours will be determined using two complementary questionnaires [102]. A revised version of the Three-Factor Eating Questionnaire (TFEQ R21) [103] will be used to assess three different domains: uncontrolled eating, emotional eating and cognitive restraint. Answers will be coded following the instruction of Cappelleri et al. [104]. The Dutch Eating Behaviour Questionnaire (DEBQ) will also be used for assessing three different domains: external, restrained and emotional eating. Answers will be coded following the instruction of Lluch et al [105]. Both questionnaire exist in French language and have been adapted to the French population [103, 105, 106].

### Ergonomic assessment

Satisfaction and adherence will be evaluated 6 months (T2) after baseline measurements.

A lack of adherence to programs aiming at increasing PA in tertiary employees has been reported multiple times [107, 108]. Genin et al. postulated that interventions targeting PA and SB in tertiary employees should not only consider the operational feasibility but also the coherence and compatibility with the working environment, structuration, functioning as well as the workers and the way they live and experience their work [24]. Therefore, an ergonomic approach is needed to improve the implementation of such an intervention in future studies but also to optimize future guidelines on strategies to reduce SB in tertiary worksite.

A satisfaction questionnaire will be given to all participants at the end of the program. A one-on-one interview will be done by an ergonomic specialist with each participant. This interview will aim to better understand (1) how PA on the workstation has been conciliated with the work tasks performed by the subject and (2) reasons of subject’s involvement in the prescription of PA. To achieve this objective, an analysis of the subject’s diary will be carried out as well as a direct, semi-direct or open interview on the participant’s motivations. These interviews will be recorded with the permission of each participant. These recordings are essential for the analysis of the verbal data collected.

### Sample size and power calculation

Sample size estimation is based on CONSORT 2010 statement for the comparison between randomized groups. In these statements, Eldridge et al. suggested “that the size of a trial should be related to the size of the future definitive RCT and for such a trial designed with 90% power and two sided 5% significance” [109]. They “recommend trial sample sizes for each treatment arm of 75, 25, 15, and 10 for standardized effect sizes that are extra small (0.1), small (0.2), medium (0.5), or large (0.8), respectively”. Considering a two-sided type I error at 5% and a statistical power greater than 80%, an effect size of 1 can be assumed for the PA and SB levels score with 18 patients per group. To take into account the lost to follow-up, a maximum of 80 patients (20 by arm, 50% females) will be included in the study.

### Statistical analyses

Statistical analyses will be conducted using Stata software (version 15, StataCorp, College Station, USA). A two-sided *p* value of less than 0.05 will be considered to indicate statistical significance. Participants will be described at baseline and between-group comparison will be tested using an unpaired *t* test for the following variables: compliance with eligibility criteria, demographic characteristics, clinical characteristics and medication. A possible difference between groups in any of these characteristics will be determined by both clinical and statistical considerations. The number of patients included and the inclusion curve will be presented by group.

The primary endpoint (time spent in PA and SB) will be compared between groups by Student’s *t* test or the non-parametric Mann-Whitney test if assumptions of parametric tests are not met. The normality will be studied using Shapiro-Wilk test. The homoscedasticity will be analysed with Fisher-Snedecor test. The results will be expressed using effect size and 95% confidence interval. Intention-to-treat analysis will be considered for the primary analysis. In order to prevent attrition bias, imputation of the missing data is planned. The statistical analysis plan also provides for an additional per-protocol analysis. Then, the analysis of the primary outcome will be completed by multivariable analysis using a linear mixed model to compare PA and SB score between randomized groups taking into account (i) covariates determined according to univariate results and to clinical relevance (gender, age and baseline PA and SB) and (ii) centre as random-effects (to measure between- and within-centres variability). The normality of the residuals from linear regression will be studied. If necessary, a logarithmic transformation of the dependent variable will be proposed. The results will be expressed using effect-sizes and 95% confidence intervals.

The comparisons between the randomized groups will be performed (1) as aforementioned for continuous secondary endpoints and (2) using the chi-squared or Fisher exact tests for categorical variables. For non-Gaussian data, results will be presented using median difference and 95% confidence intervals estimated using quantile regression model. For categorical parameters, the results will be expressed using absolute differences and 95% confidence intervals. The multivariable analysis associated to dichotomous endpoints will be generalized linear mixed model, with logit link function, and centre as random-effect. As secondary endpoints will be exploratory, no type I error correction will be applied, but the results will be expressed with odds ratios and 95% confidence intervals.

Longitudinal data will be analysed using random-effects regressions, modelling between and within patient effect, as random-effect, in addition to centre effect. The following fixed effects will be studied: randomization group, time-points evaluation and their interaction.

According to clinical relevance and to the European Medicines and Consolidated Standards of Reporting Trials recommendations, planned subgroup analyses will be proposed after completion of the study. *Subgroup × randomization group* interaction will be added to the in regression models with factors such as gender or age used for the subgroup analysis.

Particular attention, mainly descriptive, will be paid to study the adherence of participants and identify barriers and challenges to their motivations and ability to perform the PA intervention. A sensitivity analysis will be performed to study the statistical nature of missing data and to determine the most appropriate approach to the imputation of missing data: maximum bias (e.g. last observation carried forward, baseline observation carried forward) or estimation proposed by Verbeke and Molenberghs for repeated data. A study of patients’ abandoning will be proposed considering this parameter as a censored data, and consequently using the Kaplan-Meier to estimate it and log-rank for the comparison between groups.

### Data management

The Innovation and Clinical Research Direction (ICRD) of the University Hospital of Clermont-Ferrand carried out the method for data management but will not have any authority or interfere in the data entry. Qualitative and quantitative data will be entered in paper-based case report forms and electronically into a database by TG. Regular verification will be conducted to ensure the accuracy of the coding and entry by members of the REMOVE team. Access to all the information regarding the study and to the database will be restricted and controlled. The electronic data forms will be kept safe in a specific computer in the research laboratory with access code needed. All paper-based form related to the trial will be kept in locked closets. While updating the database, a complete back-up will be executed twice weekly. These back-ups will be stored on two external hard drives kept either off-site and among the research laboratory.

### Monitoring

#### Coordinating centre

The coordinating team will meet regularly, every 2 months, being composed of the principal investigator and the co-investigators. The principal investigator will supervised and coordinated the co-investigators during the trial.

#### Data monitoring committee (21a)

ICRD has also the role of the data monitoring committee. The committee may ask at any moment of the trial a report to the data management team to ensure the validity and the safety of the data. The committee will not review or discuss data pertaining to trial progress and have no competing interests.

#### Harms (22)

We do not anticipate any harm in this study. While the intervention aims to increase physical activity with very low exercise intensity, no unexplained adverse events are expected. However, if any adverse events occur during the intervention, the principal investigator will be informed. Regarding the magnitude of the harms, the principal investigator might inform the sponsor and terminate the study.

#### Auditing (23)

The sponsor might regularly audit the process of the whole study to ensure that all ethical rules are respected. An audit procedure will be executed at the end of inclusion to ensure the validity of the written informed consents. Another one will be performed after the first measurements (T0) to ensure that case report forms have been correctly fulfilled. There will not be any other auditing conducted during the intervention program, based on the low-risk intervention of this study.

### Ethics and dissemination (24–31c)

#### Ethics

Ethics approval and consent to participate have been obtained from the ethical authorities (Comité de Protection des Personne Ile de France VIII 19 09 66). Written, informed consent will be obtained from all participants.

Any modification to the protocol that affects trial methodology and outcomes will require a formal amendment. The amendment will be agreed upon by the principal investigator and has to be approved by the ethics committee.

#### Consent and ancillary studies

One of the study medical investigators (under the supervision and coordination of the principal investigator) will meet individually every subjects that expressed their willingness to participate in the present study. During the screening visit, participants will sign a written informed consent to validate their inclusion. Every participant will provide written consent for the possibility of ancillary studies. Regarding this disposition, there will be no additional consent for this study.

#### Confidentiality

All participants’ information will be collected in a pseudonymous form to ensure a complete anonymity of their data throughout the research process. Participants will be informed of the confidentiality and the anonymity of their data in the information note and the ethical application. Furthermore, participants will take notice of their ability of withdrawal from the study and to withdraw/amend parts of their data at any moment.

Data will be kept safe in a specific computer in the research laboratory with access code needed. Only the principal investigator will be allowed to open the specific session for data access.

#### Dissemination

The results of the REMOVE trial will be disseminated to participants and the collaborating tertiary societies. The study findings will be presented at national and international conferences. The results will also be submitted to peer-review journals regardless of the magnitude or direction of the outcomes.

Authorship rules will be respected based on the International Committee of Medical Journal Editors recommendations.

## Discussion

Recent literature has recognized time spent in SB and levels of PA as major predictors of cardiometabolic risks [3, 4]. With the “tertiarisation” of our societies, the worksite has become a place where SB have significantly increased [110]. It is also considered as a new strategic setting to conduct public health interventions aiming at reducing time spent sedentary. Using the constrained time employees spent seated to promote body motion appears as a promising alternative to fight against SB. In addition to work-induced sedentariness, job stress can also increase the risks of coronary heart disease [111], chronic diseases [112] and sick leave [113]. Both PA and SB are associated with decreased and increased anxiety, respectively [68, 69].

The REMOVE study will tend to provide new insight in a field that remains poorly explored [16, 25] with a potentially huge impact on global health. While the use of active desks has been mainly tested in individuals with overweight or obesity [17], the REMOVE study will determine the impact of active desk—PPM—on PA behaviour and health outcomes in healthy employees during 3 and 6 months using a primary prevention approach. The objective assessment of the effects of worksite PPM on PA and SB will be extended to the entire day and week, including non-working days. To our knowledge, this trial will be the first one to assess the medium and long-term effects of the use of cycling desk (PPM) on the physical, cardiometabolic, psychological and overall health of tertiary employees.

The literature has shown that active workstations can help to reduce SB and increase PA at work. Implementation of treadmills or cycling desks remains limited due to their high cost, large size and their lack of practicality for some population [25, 114]. Standing desk has not been shown to be a very efficient strategy as it may increase musculoskeletal risk [115] and venous insufficiency [116] without any change in energy expenditure [117]. The strength of the present study is the use of PPM device. PPM can be easily settled under a desk, spares a lot of place, is easy to use and allows any worksite to potentially implement this PPM in their offices.

Recent evidence associated LPA with a significant decrease in total and cardiovascular mortality [26]. Cardiometabolic health and especially glycaemic control are improved with LPA [118]. On top of that, multiple short periods of PA (< 10 min) have been shown to be more attractive for the sedentary populations than longer continuous bouts, while they still significantly improve physical fitness [119, 120]. Accumulated and continuous exercises have shown to elicit similar effects on physical fitness, cardiovascular and cardiometabolic parameters in inactive and sedentary populations [121, 122].

While the actual literature in this field has been shown to suffer from the use of subjective measurements and from a high methodological heterogeneity, the REMOVE project will use objective methods, which will provide with robust data. Our design, proposing two 3-month phases is also a strength of the present work as it will give the opportunity to evaluate the medium and long-term effects of the intervention, as well as key information on the long-term adherence of office workers.

Moreover, implementing PPM in multiple sites will avoid any cluster effect and reinforce the generalizability of our results [123].

Our team has previously described an important dropout rate during worksite programs that proposed on-site PA during non-working hours among office workers [108]. By contrast, this study is an interventional study, and subjects will be monitored and motivated individually every day by receiving reminders (if time spent cycling < 60 min/day) and an on-site personal visit each week. The purpose of these intervention components is to decrease dropout rate and help subjects to modify their behaviours.

Another strength of the REMOVE project is the implication of the companies’ management teams, as recently suggested [24]. Every period of experimental measures (T0, T1, T2) will last about 2 h. Measurement times will be taken on working hours and employers have accepted to pay every participant during the study visits. It is essential that participants receive financial and psychological support from their direction to be fully engaged in the research project [124]. Based on the available evidence, we are convinced of the positive effect of this protocol and its future long-term implementation within companies.

Finally, the REMOVE project proposes an innovative approach through its multidisciplinary structure by combining physiological, psychological and ergonomic assessment. Cardiometabolic parameters and psychological well-being are frequently questioned in studies focusing on tertiary employee’s health. The REMOVE project is going one-step further by integrating the ergonomic assessment to the study.

It is important to understand why our intervention can be effective but also to identify the potential barriers and challenges that can limit adherence to the proposed program. This will be assessed in the ergonomic component of the study. It will evaluate the impact of the trial on office workers and see the strengths and weaknesses of this implementation in tertiary societies. The ergonomic evaluation will also observe how the PPM will change the workspace and the quality of work. This assessment is crucial to allow further implementation in companies.

At the end of the study, all employees (participants and non-participants) and managers will have a feedback on the study results. The expected positive results will motivate employers to implement PPM in their enterprises but also employees to start or continue this type of PA, perhaps in addition to more structured PA outside of work [124].

In conclusion, this study has substantial interest because the tertiary sector represents between 60 and 80 % of workers in western countries [125]. It is time to stop focusing on treatment and disease management and to shift our efforts towards the prevention of the risks of preventable diseases, especially those associated with large volumes of SB. If our hypotheses are confirmed, results from our study will have important clinical, scientific but also societal implications. It will open new venues for future public health policies to protect people from the development of cardiometabolic disorders related to their occupation.

### Trial status

Specific societies with tertiary employees have been contacted since January 2020. The study was planned to start in September 2020, but due to COVID-19, the recruitment phase has been delayed. Finally, recruitment finished on 15 March 2021. The study is anticipating to be completed on September 2021. This version is V1-07-08-2020.

## Data Availability

The principal investigator will be given access to the full data sets. The datasets analysed during the present study may be shared from the corresponding author on reasonable request with the approval of the principal investigator and the sponsor. The French model consent form is available from the corresponding author on request.

## Abbreviations

AEA: N-arachidonylethanolamine
AG: Actigraph GT3x-BT
AP: ActivPAL3 micro
EQUA-S: The Two Factor Equanimity Scale
ERI: Effort-reward imbalance
FFM: Fat-free mass
FM: Fat mass
METs: Metabolic equivalents
NMQ: Nordic Musculoskeletal Questionnaire
PA: Physical activity
PPM: Portable pedal machine
RMR: Resting metabolic rate
RPAQ: Recent Physical Activity Questionnaire
SB: Sedentary behaviours
VAS: Visual analogue scale
WHtR: Waist to height ratio
1-RM: One-repetition maximum
